# Supplementary material for: Mapping multimorbidity progression among 190 diseases
Source: Commun Med (Lond). 2024 Jul 11;4:139. doi: 10.1038/s43856-024-00563-2 (PMC11239867; doi:10.1038/s43856-024-00563-2)
Supplement: Supplementary file 2 — Supplementary materials [file 43856_2024_563_MOESM2_ESM.pdf]

Supplementary Information for "Mapping multimorbidity progression among 190 diseases"

Shasha Han[1,2,3*], Sairan Li[1], Yunhaonan Yang[4], Lihong Liu[5], Libing Ma[6], Zhiwei Leng[7], Frances S Mair[8], Christopher R Butler[9,10], Bruno Pereira Nunes[11,12], J. Jaime Miranda[13,14], Weizhong Yang[1,2,3], Ruitai Shao[1,2,3], Chen Wang[1,2,3,15*]

1. School of Population Medicine and Public Health, Chinese Academy of Medical Sciences & Peking Union Medical College, Beijing, China.
2. State Key Laboratory of Respiratory Health and Multimorbidity, Beijing, China,100730
3. Key Laboratory of Pathogen Infection Prevention and Control (Peking Union Medical College), Ministry of Education, Beijing, China.
4. Section of Epidemiology and Population Health, West China Second University Hospital, Sichuan University, Chengdu, China.
5. China-Japan Friendship Hospital, Beijing, China.
6. Affiliated Hospital of Guilin Medical University, China.
7. Peking Union Hospital, China.
8. School of Health and Wellbeing, College of Medicine, Veterinary and Life Sciences, University of Glasgow, Glasgow, Scotland, UK.
9. Department of Brain Sciences, Imperial College London, London, UK.
10. Imperial College Healthcare NHS Trust, UK.
11. Postgraduate Program of Nursing, Federal University of Pelotas, Pelotas, Brazil.
12. Postgraduate Program of Epidemiology, Federal University of Pelotas, Pelotas, Brazil.
13. Sydney School of Public Health, Faculty of Medicine and Health, University of Sydney, Sydney, Australia.
14. CRONICAS Centre of Excellence in Chronic Diseases, Universidad Peruana Cayetano Heredia, Lima, Peru.
15. Chinese Academy of Medical Sciences & Peking Union Medical College, Beijing, China.

[*] **Corresponding Authors**:
Shasha Han, PhD, Assistant Prof.
School of Population Medicine and Public Health
Chinese Academy of Medical Sciences & Peking Union Medical College
Beijing, P. R. China. 100006
hanshasha@pumc.edu.cn

Chen Wang, MD, Prof.

Chinese Academy of Medical Sciences & Peking Union Medical College
Beijing, P. R. China. 100730
wangchen@pumc.edu.cn

Table of Contents

Supplementary Methods 4
Sample 4
Ethics approval 4
Measures 4
Statistical analysis 5
Pairwise causal effects 5
Key diseases in multimorbidity progress 7
Clustering consequential spectrum rays and clustering causal spectrum rings 8
One-step multimorbidity progress pairs 8
Clustering one-step multimorbidity progress 9
Supplementary Results 10
Top influenced diseases 10
Clustering consequential spectrum rays and causal spectrum rings reveal shared multimorbidity development mechanisms 11
Supplementary Figures 13
Supplementary Figure 1: Heatmap chart showing the clustering of diseases' consequential spectrum rays 14
Supplementary Figure 2: Heatmap chart showing the clustering of diseases' causal spectrum rings. 15
Supplementary Figure 3: Dendrogram showing the clustering of diseases' consequential spectrum rays and rings. 16
Supplementary Figure 4: Illustrations of the one-step multimorbidity progress within and across ICD-10 chapters. 18
Supplementary Figure 5: Illustrations of the one-step multimorbidity progress across ICD-10 chapters only. 20
Supplementary Figure 6: Median number of years between the onset of diseases studied. 23
Supplementary Figure 7: Distributions of the median number of years between the onset of top diseases and other diseases 24
Supplementary Figure 8: Changes in pairwise causal effects between the main analysis and the secondary analysis with one year of follow-up. 26
Supplementary Figure 9: Venn diagrams show the overlap of identified pairs with significant causal effects between the main analysis and merged scenario. 27
Supplementary Tables 28
Supplementary Table 1. Detailed baseline variables in UKBB 28
References 29

Supplementary Methods

Sample

The UKB is a multicenter, community-based cohort study conducted in the United Kingdom from March 2006 to December 2010 for 502,413 participants at baseline, of whom 54.4% were women (aged 37 to 73 years, mean[SD] age 57.1[8.1] years). All participants were registered with the UK National Health Service and attended an initial examination, and were followed up longitudinally through linkage to electronic health-related records. Ongoing inpatient hospital records beginning in 1995 until September 2021 were used to identify diagnoses according to the International Statistical Classification of Diseases and Related Health Problems, Tenth Revision (ICD-10) codes. End of follow-up was defined as end of hospital inpatient data collection in September 2021; mean longitudinal follow-up was 12.7 [0.9] years. Participant written informed consent was obtained prior to data collection.

Ethics approval

This study was covered by the ethical approval from the UKB granted by the National Information Governance Board for Health and Social Care and the NHS North West Multicenter Research Ethics Committee. All participants provided informed consent through electronic signature at baseline assessment. Ethical approval of the study was obtained from the Chinese Academy of Medical Sciences & Peking Union Medical College. All data extracted were deidentified for analysis.

Measures

<u>Disease status</u>

Disease status was obtained from participants' hospital inpatient records and coded according to the third ICD-10 level. A total of 1,938 diseases, classified by the ICD-10 code with one-letter and two-digit numbers, were recorded in the UKB dataset. Diseases related to malformations, and abnormal findings (Q00–Q99, R00–R99), external causes (S00–T98, V01–Y98), and factors influencing health status and contact with health services (Z00–Z99) were excluded. For analysis, we selected the diseases that had prevalence rates larger than 1% for females and males separately, which comprised 154 distinct diseases for females and 160 distinct diseases for males.

The study involved 16 ICD-10 chapters, infectious and parasitic diseases (A00-B99),neoplasms (C00-D48), diseases of the blood and blood-forming organs and disorders involving the immune mechanism (D50-D89), endocrine, nutritional and metabolic diseases (E00-E90), mental and behavioural disorders (F00-F99), diseases of the nervous system (G00-G99), diseases of the eye and adnexa (H00-H59),

diseases of the ear and mastoid process (H60-H95), diseases of the circulatory system (I00-I99), diseases of the respiratory system (J00-J99), diseases of the digestive system (K00-K93), diseases of the skin and subcutaneous tissue (L00-L99), diseases of the musculoskeletal system and connective tissue (M00-M99), diseases of the genitourinary system (N00-N99), pregnancy, childbirth and the puerperium (O00-O99), codes for special purposes(U00-U85).

Baseline covariates

Sociodemographic factors (age, sex, race, education level, deprivation, social activities), health behaviors (smoking, alcohol use, sleep duration, phone use, physical activity, gas use), health status (body mass index, systolic blood pressure, diastolic blood pressure, glycated hemoglobin, high-density lipoproteins, low-density lipoproteins, triglycerides, calcium, C-reactive protein, and long-illness diseases), doctor-diagnosed chronic diseases, and family history (ages of mother's and father's death and illnesses of mother and father) were included in baseline covariates to control for measured confounding. Additionally, all disease status and factors influencing health status and contact with health services (ICD codes, Z00–Z99) at baseline were included to control confounding. Factors influencing health status and contact with health services include ICD-10 codes for Persons encountering health services for examinations, Genetic carrier and genetic susceptibility to disease investigation, for specific procedures and health care, in other circumstances; Persons with potential health hazards related to communicable diseases, socioeconomic and psychosocial circumstances, family and personal history, certain conditions influencing health status, etc.

Townsend Deprivation Index was used as a measure of deprivation, which combines information on housing, employment, car availability, and social class, with higher values indicating greater deprivation. Physical activity level was measured by the International Physical Activity Questionnaire (IPAQ) and was stratified into three levels: low, medium, and high. For the measure of other covariates, see Supplementary Table 1.

Statistical analysis

Pairwise causal effects

We used the target maximum likelihood estimation (TMLE) method to estimate the effect of exposure to one disease on the risk of another condition. [1] TMLE is a doubly robust maximum-likelihood–based approach that includes a secondary targeting step for optimizing the bias-variance tradeoff for the target parameter. Specifically, it was conducted following the four steps:

Step 1: We estimated the expected models using the logistic models, with all baseline covariates and exposure disease status as independent variables.

Step 2: We estimated the propensity scores models using the logistic models, with all baseline covariates status as independent variables, which were then used to build the fluctuation parameters, which are the inverse probability of exposure minus the inverse probability of unexposure.
Step 3: We updated the expected outcome models from **Step 1** by fitting a logistic regression using the fluctuation parameters estimated from **Step 2** as the only predictor and the initially expected outcome under the observed exposure as a fixed intercept.
Step 4: We calculated the average difference between the updated expected outcomes from **Step 3**, under the two exposure conditions.

We conducted the estimation for females and males separately, resulting in a comprehensive assessment covering 23,562 directional pairwise causal effects of diseases (154 x 153) for females and 25,440 directional pairwise causal effects of diseases (160 x 159) for males. For each directional pair of diagnoses, we assessed how the presence of the disease affects the likelihood of developing the other disease. Patients with a history of the two diseases before baseline or with the outcome disease occurring before the exposure disease were excluded from the estimation. Supplementary Data 4 and 5 provide details on the number of samples and follow-up years for each disease pair. For those who developed the exposure disease and subsequently the outcome disease during the study period, Supplementary Data 4 and 5 also present the samples and the number of years between the onset of the two diseases. Most of them (females, 71.3%; males, 69.7%) had a median gap of 1 to 5 years, a considerable number of them (females, 26.6%; males, 28.9%) had median gaps of less than 1 year, while only a small percentage (females, 2.1%; males, 1.4%) had a gap exceeding 5 years (Extended Data Fig. 6).

To control for measured confounding, [57] we included all baseline covariates, including all other disease statuses beyond the two under investigation and factors influencing health status and contact with health services (Z00–Z99) at baseline, in the TMLE estimation. These factors can be considered as proxy indicators that reflect hospital access and other existing health conditions. The variables had missing percentages ranging from 0.0% to 19.9%, with the exception of the mother's and father's ages of death, which had missing percentages of 41.7% and 26.9%, respectively. In the main analysis, missing data were imputed once by chained equation. [2] A sensitivity analysis was conducted to investigate whether the main conclusions remained after accounting for variations across different imputations. In the sensitivity analysis, missing values were imputed using the multiple imputation method with 5 imputed data sets. Estimation with TMLE was done for each imputed data set and was merged using the Rubin rule. [3] P values were subsequently adjusted for multiple testing based on false discovery rate using Benjamini and Hochberg method. We compared the number of significant causal effects (adjusted P values < 0.01) for the main analysis and the merged scenarios. The results showed that over 97% of the causal pairs were identified in both methods, for both females and males (Supplementary Figure 9). Furthermore, the pairs identified in only one scenario had effects close to zeros.

Violations of positivity assumptions could lead to extreme values in the estimated propensity score. We followed the common approach and trimmed individuals with propensity scores outside a common range, which is formed by the lowest propensity scores in the treated (having the exposure disease) individuals and the highest propensity scores in the control individuals (not having the exposure disease). [4] Propensity scores were reestimated after the trimming, and the TMLE method was redone. In cases where propensity scores fell below 0.01 or above 0.99, we performed a second trimming using the cutoffs of the 5th percentile in the treated individuals and the 95th percentile in the control individuals. [4]

In order to assess the rapidity of disease impact, we limited our analysis to a one-year follow-up. This approach, however, resulted in a much smaller set of exposure-outcome disease pairs to be examined since individuals who developed the exposure diseases after one year were excluded. To overcome this limitation, we expanded our investigation to include all patients who had developed the exposure disease, and we tracked them for one year. Subsequently, we carried out a sensitivity analysis using the same estimation method.

Key diseases in multimorbidity progress

To identify the key diseases in multimorbidity progress, we compared the consequential spectrum rays and causal spectrum rings of diagnoses introduced earlier. We categorized the causal effects based on their magnitude. Effects with an absolute value larger than 0.01 were considered strong, while others were classified as weak. We estimated the proportions of strong increase (effect size $\geq$0.01), strong reduction (effect size $\leq$ -0.01), weak increase ($0 <$ effect size < 0.01), and weak reduction ($-0.01 <$ effect size < 0) in each consequential spectrum ray and each causal spectrum ring. Using these proportions, we ranked the diseases according to four criteria: (1) the extent to which they strongly increase other diseases, (2) the extent to which they strongly reduce other diseases, (3) the extent to which other diseases strongly increase them, (4) the extent to which other diseases strongly reduce them. Diseases that scored high on criteria (1) and (3) were identified as critical diseases in the progression of multimorbidity. Conversely, diseases that scored high on criteria (2) and (4) were identified as crucial diseases in the prevention of multimorbidity, whereby exposure to one disease reduces the likelihood of another disease occurring.

To better understand the rapid progression of multimorbidity, we analyzed the median number of years between the onset of key diseases and other diseases. We used data from individuals who experienced these key diseases and other diseases during the study period. For each key influential disease, we estimated the distribution of the median number of years between their onset and the emergence of subsequent diseases along the consequential spectrum ray; for each key influenced

disease, we estimated the distribution of the median number of years between the emergence of previous diseases and their onset along the consequential spectrum ring.

Clustering consequential spectrum rays and clustering causal spectrum rings

We analyzed consequential spectrum rays to identify groups of diseases that share common patterns of consequential effects. To do this systematically, we used the Ward's hierarchical clustering algorithm to group diseases based on the similarities in their consequential spectrum rays. [5,6] We dichotomized rays and assigned 1 for the increasing effects, -1 for the reducing effects, and 0 otherwise to identify signs of rays that indicate the presence of a multimorbidity progression. The similarity between two consequential spectrum rays was determined by the correlation (r) between the two dichotomized rays across all diseases, and was converted to squared distance in Euclidean space through $1 - r$. We performed the clustering separately for females (154 rays) and males (160 rays). Identically, we clustered causal spectrum rings to identify groups of diseases with similar patterns of causal mechanisms separately for females (154rings) and males (160 rings). Self-to-self effect was set as 1 in the analysis.

We assessed the significance of clustering using the approximately unbiased test that is based on the multiscale bootstrap technique and tends to be less biased when assessing the confidence of clustering. [7] Clustering confidence is reflected by P_{au}, where the greater the P_{au}, the greater the probability that the identified network is true.

We created clusters according to the vertical lines intersected by the threshold line and P_{au} in the dendrogram. The threshold was determined by fixed heights of the dendrograms (ray clustering 0.661, ring clustering 1.260), chosen to better differentiate cluster patterns. If a cluster formed by the threshold has a P_{au} less than 0.40, we instead used its child clusters (Supplementary Figure 3).

One-step multimorbidity progress pairs

We considered the one-step multimorbidity progress between pairs of diseases (such as D1 and D2), denoted by D1 ⇔ D2. All pairs could be classified into three groups, with one group of pairs having increasing effects in both directions (bi-increasing, D1 $\xrightarrow{+}$ D2, D1 $\xleftarrow{+}$ D2), one group of pairs having increasing effects in one direction and non-increasing effects in the other direction (uni-increasing, D1 $\xrightarrow{+}$ D2, D1 $\xleftarrow{-/0}$ D2), and one group of pairs having non-increasing effects in either direction (non-increasing, D1 $\xrightarrow{-/0}$ D2, D1 $\xleftarrow{-/0}$ D2). Patients diagnosed with one of the bi-increasing progress pairs would have a higher likelihood of developing other diseases and experiencing multimorbidity. In contrast, patients diagnosed with one of the non-increasing pairs would not appear to be more prone

to developing other diseases. In cases of uni-increasing pair, patients diagnosed with the starting diagnosis (D1) in the direction of the increasing effect (D1 $\xrightarrow{+}$ D2) may progress into multimorbidity, but not in the opposite direction (D1 $\xleftarrow{-/0}$ D2).

We focused on one-step multimorbidity progress where one disease can strongly increase the occurrence of another. For females, we found 1553 (65.0%) pairs of bi-directional and 1080 (45.9%) pairs of uni-directional progress with effect sizes larger than 0.01. For males, we found 1675 (68.6%) pairs of bi-directional and 1339 (50.4%) pairs of uni-directional progress with effect sizes larger than 0.01. Bi-directional progress had effect sizes larger than 0.01 on both sides. We then further analyzed these strong bi-directional and uni-directional progressions of multimorbidity.

To investigate which pairs of ICD-10 chapters commonly associated with multimorbidity, we counted the number of links for each pair of 16 ICD-10 chapters involved in the study, including chapters paired with themselves. To identify significant chapter pairs, we used a permutation test. We shuffled the links 10^4 times while keeping the node degree the same, and counted the number of links for each randomization. With this information, we calculated an empirical P value, P_{perm}, for each chapter pair to evaluate their significance.

$$P_{perm}^{(ab)} = \frac{\sum_{i=1}^{10^4} 1_{\{C_{ab}^{i} \geq C_{ab}^{obs}\}}}{10^6},$$

where the indicator $1_{\{C_{ab}^{i} \geq C_{ab}^{obs}\}}$ is 1 if the number of links between the chapter pairs *a* and *b* in the *i*th randomization (C_{ab}^{i}) is equal or larger than the observed links between the chapter pair (C_{ab}^{obs}), and 0 otherwise. The analysis was done separately for bi-directional and uni-directional progress, and for females and males.

Clustering one-step multimorbidity progress

We clustered one-step multimorbidity progress into multimorbidity progress constellations consisting of more than two diseases. To do so, we constructed a graph where each disease was represented by a node, and pairwise connections between them were represented by edges, with a value of 2 for bi-directional progress pairs, 1 for uni-directional progress pairs, and 0 otherwise. We then partitioned the graph using a self-tuning k-means clustering method that generates 1000 clustering solutions and selects the one with the minimum total within-cluster sum of squared distances as the final solution. Finally, we regrouped the isolated disease nodes into their mostly connected clusters based on the number of links to diseases in each cluster. The number of clusters was determined by the average silhouette method.[8]

To assess the stability of our clustering, we compared it with 10^4 clusterings from traditional k-means method with different initial random seeds. The similarity between the clusterings was measured using the adjusted Rand index, which calculates the probability of the two clusterings agreeing on a randomly selected pair of nodes after accounting for chance. [9] We averaged the 10^4 adjusted Rand index, and a higher value indicates greater stability in our clustering results.

As an alternative sensitivity analysis, we tested the stability by comparing results across distinct clustering methods. Instead of using k-means clustering, we partitioned the graph using a self-tuning spectral clustering method that uses the adaptive density-aware kernel to strengthen connections in the graph based on common nearest neighbours. [10,11] The complete procedure involved transforming the graph into an affinity matrix using the Gaussian kernel, generating a matrix with eigenvectors, and carrying out K-means clustering. Identically, we measured the similarity between the clusterings and ours using the adjusted Rand index. We found that the adjusted Rand Indexes were still sufficiently high, 0.643 and 0.679 for clusterings in females and males respectively.

We used the Kamada-Kawai algorithm to create a visually appealing cluster visualization of disease nodes by placing more strongly connected nodes in close proximity and minimizing edge crossings. [12] To determine the hub disease within each cluster, we employed the PageRank algorithm for each constellation. [13] We emphasized the direction and strength of progress and weighted the directional links using the estimated pairwise causal effects. Bi-redirectional progress were considered in two directions separately. The algorithm assessed the quality and quantity of links to each node, providing a reliable estimate of their overall importance.

Supplementary Results

Top influenced diseases

We identified the top 10 diseases with the highest proportion of strong reduction in the consequential spectrum rays for both males and females (Fig. 1b). Six of these diagnoses were common to both sexes. The top 1 in both females and males was disorders of refraction and accommodation (H52). Other five diagnoses were malignant neoplasm (C34, C44, C78, and C79) and haemorrhoids and perianal venous thrombosis (K64). Both female and male patients starting with these diagnoses were less likely to develop other diagnoses. It was noted that the new diseases of uncertain etiology or emergency use (U07) in males were less likely to reduce many other diseases except several bacterial infections (A09, A41, B96), purpura and other haemorrhagic (D69), several metabolic disorders (E83, E86, E87), delirium (F05), hemiplegia (G81), several circulatory diseases (I26, I67, I95), several respiratory diseases (J18, J43, J90, J96, J98), functional intestinal disorders (K59), decubitus ulce (L89), acute renal failure (N17).

We further identified the top 10 diseases with the highest proportion of strong reduction in the consequential spectrum rays for both males and females (Fig. 1d). The top 1 in both females and males was haemorrhoids (I84), indicating that both female and male patients with a wide range of different diagnoses were less likely to develop haemorrhoids in the current clinical practice, compared to those without the anterior diagnoses. Also, internal derangement of knee (M23), cataract (H26), anus and rectum (K62), gonarthrosis (M17) and malignant neoplasms of skin (C44) were less likely to be induced by other diseases in both sexes. Both female and male patients starting from different diagnoses would be less likely to progress into multimorbidity with these diagnoses. Additionally, we discovered that malignant neoplasms of skin (C44) had strongly reduced many other diagnoses, as well as being greatly reduced by many other diagnoses in both females and males.

Clustering consequential spectrum rays and causal spectrum rings reveal shared multimorbidity development mechanisms

When using consequential spectrum rays to cluster diseases, we identified 26 clusters for females and 28 clusters for males (mean clustering significance, female P_{au} = 0.87, male P_{au} = 0.88, Fig. 3). We identified several isolated clusters, each containing only one disease. In females, they included the ones related to labor and delivery (V and VIII), nose issues and nasal sinus disorders (XX), and benign lipomatous neoplasms (XXV). Apart from the childbirth-related clusters, the other single-disease clusters in females were associated with diseases that did not pose a significant risk for most other disease conditions. The single-disease clusters in males were the hypertension cluster (I), as well as clusters related to retinal disorder (XV) and benign neoplasm of skin (XXVIII). All of these clusters, except for skin cancer, were associated with diseases that increased the risk of developing most disease conditions.

We further observed a notable difference in hypertension (I10) between males and females. In males, it appeared distinct while in females, it was grouped with asthma (J45). However, it is worth noting that this clustering of the diseases using consequential spectrum rays can only capture their impact on other diseases, not the other way around.

In addition to providing important insights on multimorbidity progress, clustering consequential spectrum rays and causal spectrum rings sheds light on how certain diseases are prevented from contributing to this progress. Diseases that either decrease the likelihood of other diseases or are themselves decreased by other diseases are less likely to be involved in multimorbidity progress. We found that the top 10 diseases that strongly decreased most other diseases (Fig. 4) and respectively were strongly decreased by most other diseases covered a broad spectrum of clusters in both sexes (Extended Data Fig. 1). This suggests that the underlying reasons for diseases' absence from

multimorbidity progress may be complex and require careful examination to understand their etiology and treatment practices.

Supplementary Figures

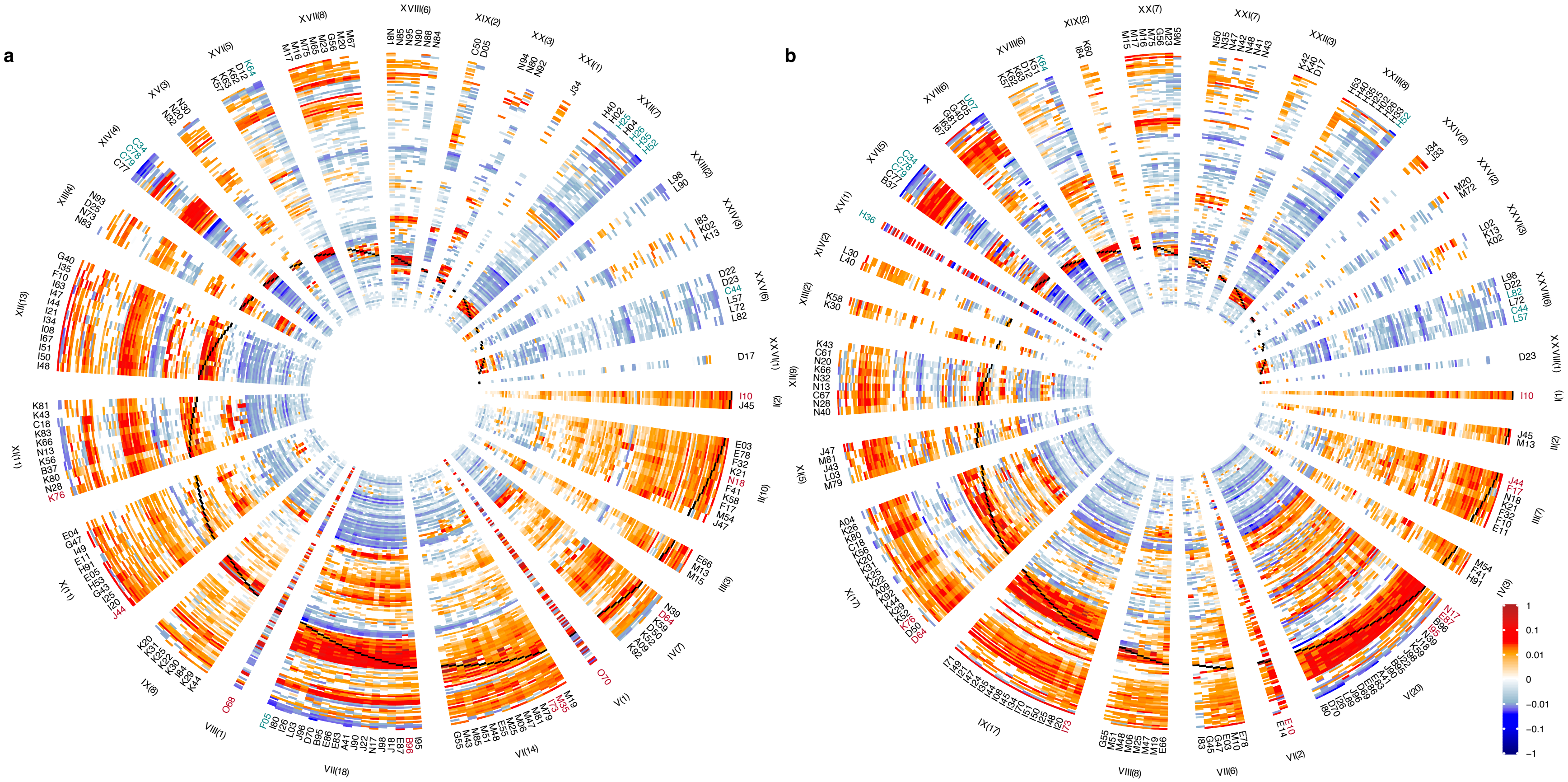

Supplementary Figure 1: Heatmap chart showing the clustering of diseases' consequential spectrum rays. a, females. **b**, males. Diseases that have similar consequential spectrum rays are clustered together and have similar patterns of affecting other diseases. The number of diseases in a cluster is indicated inside the parentheses. Identified top influential and influenced diseases affecting others in **Figures 3a** and **3b** are denoted in red and green respectively. Diseases names of these top influenced codes can be found in in **Figures 3a** and **3b.** Disease names of other ICD-10 codes can be found in **Figures 1** and **2**.

a

b

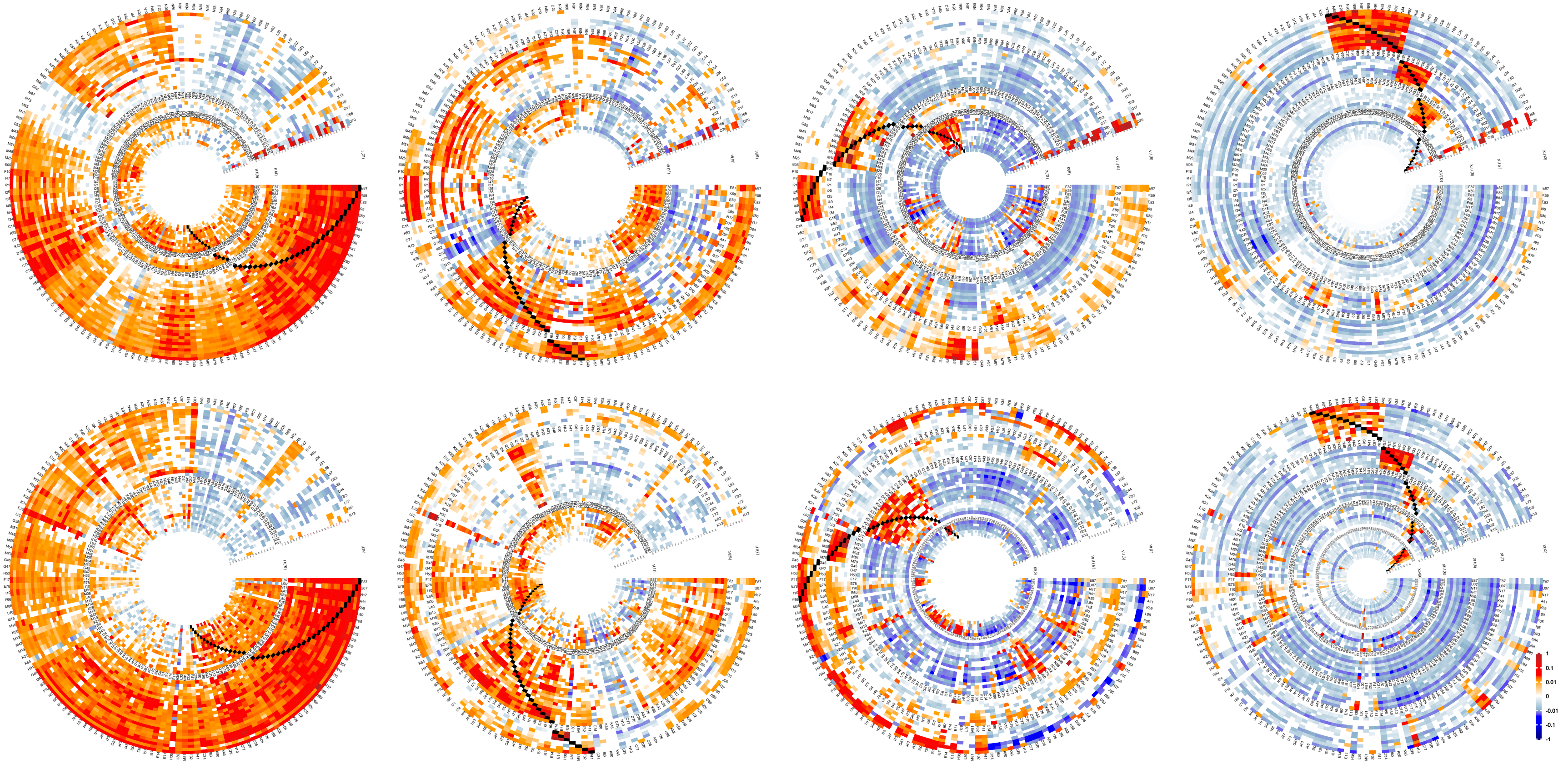

Supplementary Figure 2: Heatmap chart showing the clustering of diseases' causal spectrum rings. a, females. **b**, males. Diseases that have similar causal spectrum rings are clustered together and have similar patterns of being affected by other diseases. Identified top diseases affecting others in **Figures 3c** and **3d** are denoted in red and green respectively**.** Diseases names of these top influenced diseases can be found in in **Figures 3a** and **3b.** Disease names of other ICD-10 codes can be found in **Figures 1** and **2**.

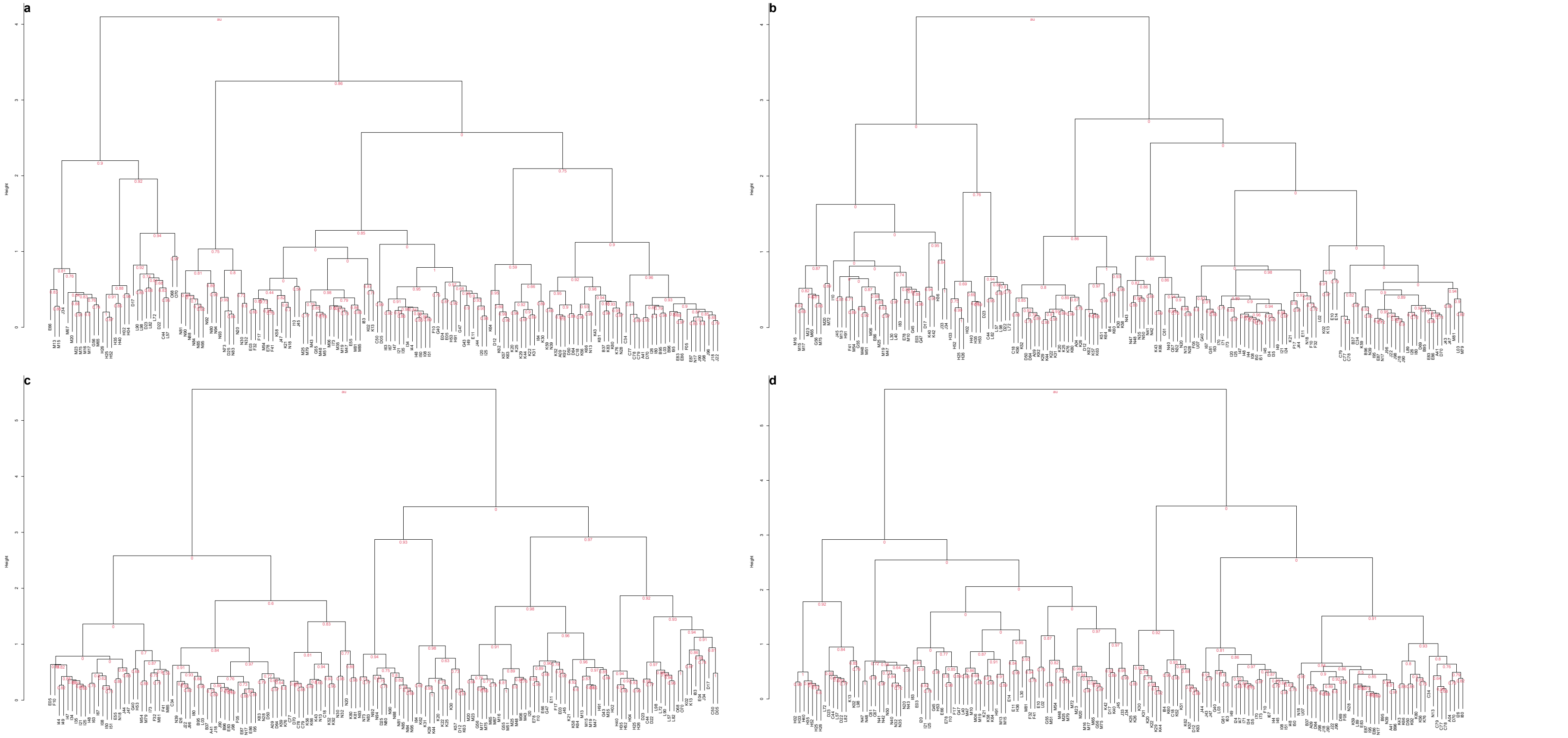

Supplementary Figure 3: Dendrogram showing the clustering of diseases' consequential spectrum rays and rings. a, females rays. **b**, males rays. **c**, females rings. **d**, males rings. Confidence is reflected by P_{au}, where the greater the P_{au}, the greater the probability that the identified network is true.

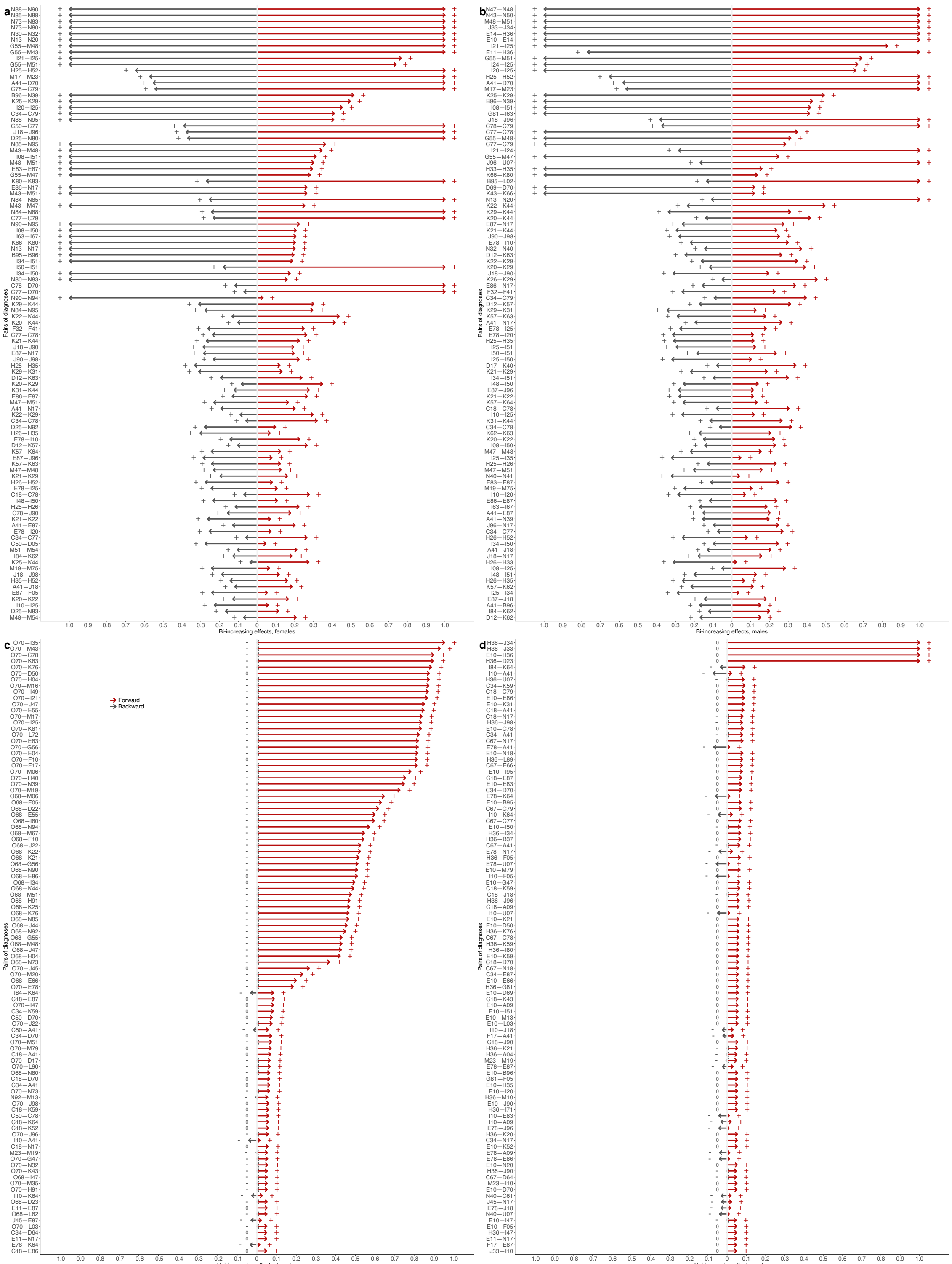

a
Pairs of diagnoses
Bi-increasing effects, females
b
Bi-increasing effects, males
c
Forward
Backward
Uni-increasing effects, females
d
Uni-increasing effects, males

Supplementary Figure 4: **Illustrations of the one-step multimorbidity progress within and across ICD-10 chapters**. **a**, females, bi-directional. **b**, males, bi-directional. **c**, females, uni-directional. **d**, males, uni-directional. Lengths of segments represent the estimated causal effects. Bi-directional progress was ordered by the sum of the causal effects in two directions. Uni-directional progress was ordered by the difference in the causal effects between the increasing direction and the non-increasing direction. Only the top 100 were displayed. 95% confidence intervals are shown in Supplementary Data 1 and 2.

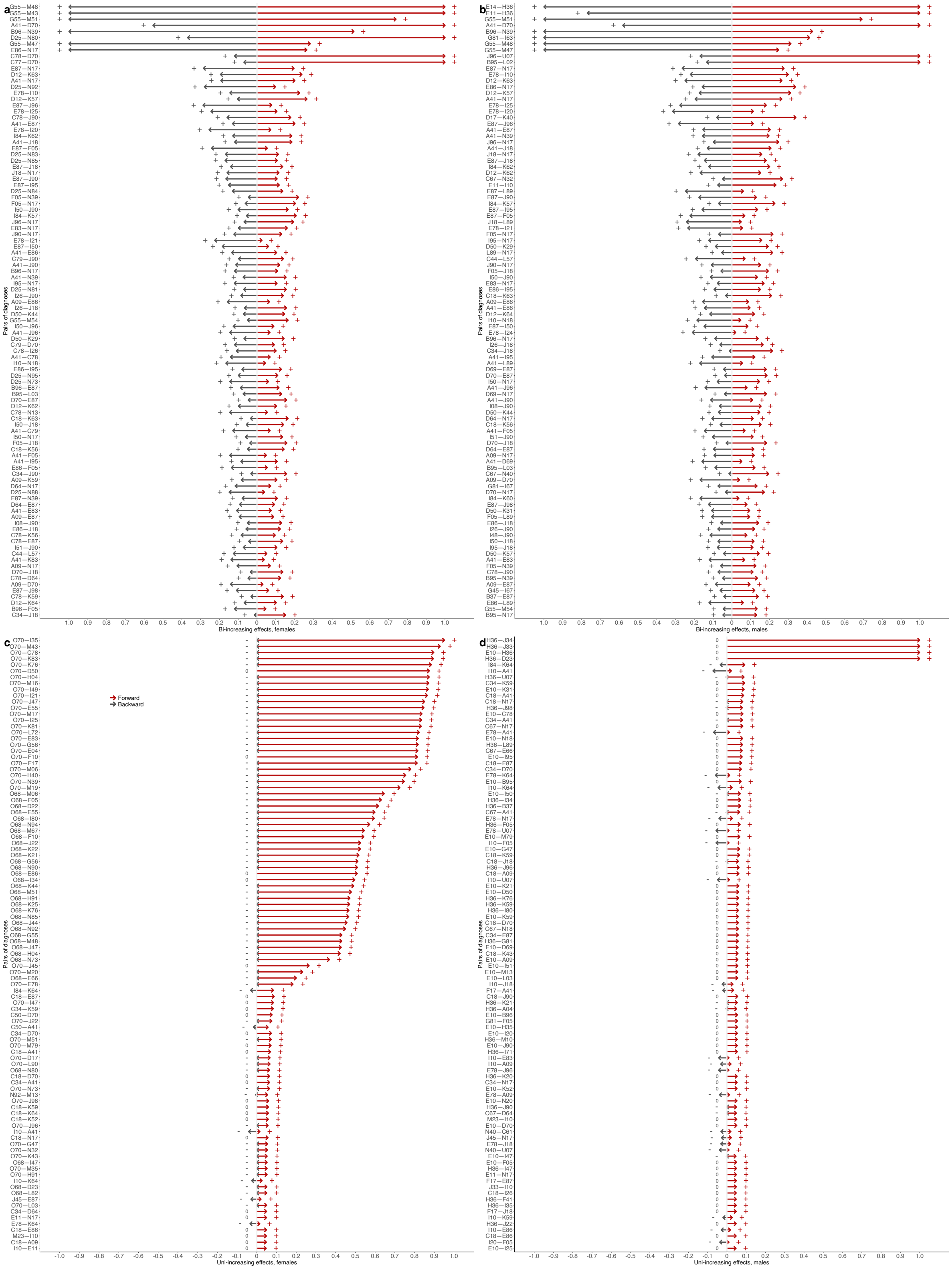
a
b
c
d
Pairs of diagnoses
Bi-increasing effects, females
Bi-increasing effects, males
Uni-increasing effects, females
Uni-increasing effects, males
Forward
Backward

Supplementary Figure 5: **Illustrations of the one-step multimorbidity progress across ICD-10 chapters only.a**, females, bi-directional. **b**, males, bi-directional. **c**, females, uni-directional. **d**, males, uni-directional Lengths of segments represent the estimated causal effects. Bi-directional progress was ordered by the sum of the causal effects in two directions. Uni-directional progress was ordered by the difference in the causal effects between the increasing direction and the non-increasing direction. Only the top 100 were displayed. 95% confidence intervals are shown in Supplementary Data 1 and 2.

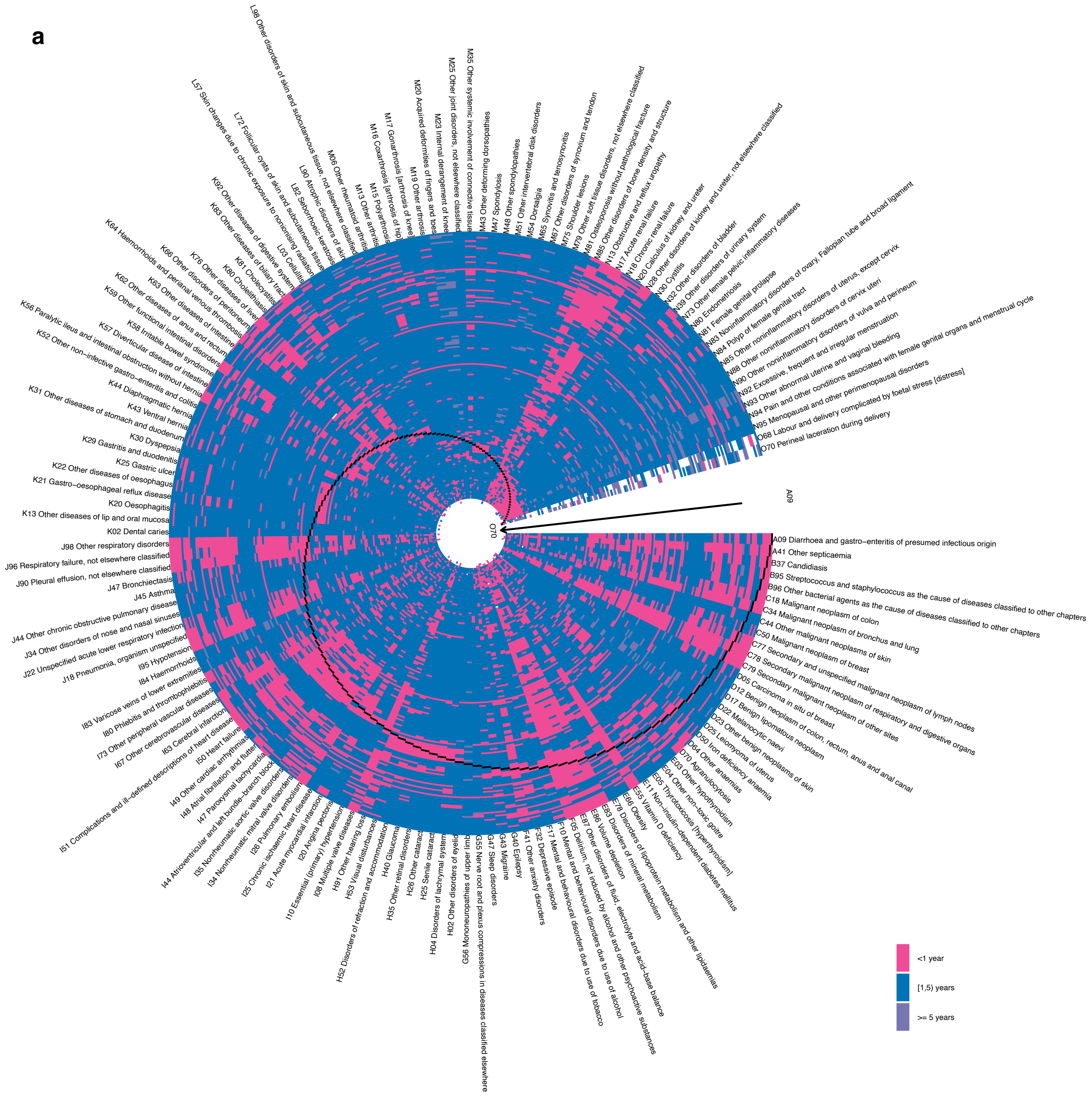
a
A09
O70
A09 Diarrhoea and gastro−enteritis of presumed infectious origin
A41 Other septicaemia
B37 Candidiasis
B95 Streptococcus and staphylococcus as the cause of diseases classified to other chapters
B96 Other bacterial agents as the cause of diseases classified to other chapters
C18 Malignant neoplasm of colon
C34 Malignant neoplasm of bronchus and lung
C44 Other malignant neoplasms of skin
C50 Malignant neoplasm of breast
C77 Secondary and unspecified malignant neoplasm of lymph nodes
C78 Secondary malignant neoplasm of respiratory and digestive organs
C79 Secondary malignant neoplasm of other sites
D05 Carcinoma in situ of breast
D12 Benign neoplasm of colon, rectum, anus and anal canal
D17 Benign lipomatous neoplasm
D22 Melanocytic naevi
D23 Other benign neoplasms of skin
D25 Leiomyoma of uterus
D50 Iron deficiency anaemia
D64 Other anaemias
D70 Agranulocytosis
E03 Other hypothyroidism
E04 Other non−toxic goitre
E05 Thyrotoxicosis [hyperthyroidism]
E11 Non−insulin−dependent diabetes mellitus
E55 Vitamin D deficiency
E66 Obesity
E78 Disorders of lipoprotein metabolism and other lipidaemias
E83 Disorders of mineral metabolism
E86 Volume depletion
E87 Other disorders of fluid, electrolyte and acid−base balance
F05 Delirium, not induced by alcohol and other psychoactive substances
F10 Mental and behavioural disorders due to use of alcohol
F17 Mental and behavioural disorders due to use of tobacco
F32 Depressive episode
F41 Other anxiety disorders
G40 Epilepsy
G43 Migraine
G47 Sleep disorders
G55 Nerve root and plexus compressions in diseases classified elsewhere
G56 Mononeuropathies of upper limb
H02 Other disorders of eyelid
H04 Disorders of lachrymal system
H25 Senile cataract
H26 Other cataract
H35 Other retinal disorders
H40 Glaucoma
H52 Disorders of refraction and accommodation
H53 Visual disturbances
H91 Other hearing loss
I08 Multiple valve diseases
I10 Essential (primary) hypertension
I20 Angina pectoris
I21 Acute myocardial infarction
I25 Chronic ischaemic heart disease
I26 Pulmonary embolism
I34 Nonrheumatic mitral valve disorders
I35 Nonrheumatic aortic valve disorders
I44 Atrioventricular and left bundle−branch block
I47 Paroxysmal tachycardia
I48 Atrial fibrillation and flutter
I49 Other cardiac arrhythmias
I50 Heart failure
I51 Complications and ill−defined descriptions of heart disease
I63 Cerebral infarction
I67 Other cerebrovascular diseases
I73 Other peripheral vascular diseases
I80 Phlebitis and thrombophlebitis
I83 Varicose veins of lower extremities
I84 Haemorrhoids
I95 Hypotension
J18 Pneumonia, organism unspecified
J22 Unspecified acute lower respiratory infection
J34 Other disorders of nose and nasal sinuses
J44 Other chronic obstructive pulmonary disease
J45 Asthma
J47 Bronchiectasis
J90 Pleural effusion, not elsewhere classified
J96 Respiratory failure, not elsewhere classified
J98 Other respiratory disorders
K02 Dental caries
K13 Other diseases of lip and oral mucosa
K20 Oesophagitis
K21 Gastro−oesophageal reflux disease
K22 Other diseases of oesophagus
K25 Gastric ulcer
K29 Gastritis and duodenitis
K30 Dyspepsia
K31 Other diseases of stomach and duodenum
K43 Ventral hernia
K44 Diaphragmatic hernia
K52 Other non−infective gastro−enteritis and colitis
K56 Paralytic ileus and intestinal obstruction without hernia
K57 Diverticular disease of intestine
K58 Irritable bowel syndrome
K59 Other functional intestinal disorders
K62 Other diseases of anus and rectum
K63 Other diseases of intestine
K64 Haemorrhoids and perianal venous thrombosis
K66 Other disorders of peritoneum
K76 Other diseases of liver
K80 Cholelithiasis
K81 Cholecystitis
K83 Other diseases of biliary tract
K92 Other diseases of digestive system
L03 Cellulitis
L57 Skin changes due to chronic exposure to nonionising radiation
L72 Follicular cysts of skin and subcutaneous tissue
L82 Seborrhoeic keratosis
L90 Atrophic disorders of skin
L98 Other disorders of skin and subcutaneous tissue, not elsewhere classified
M06 Other rheumatoid arthritis
M13 Other arthritis
M15 Polyarthrosis
M16 Coxarthrosis [arthrosis of hip]
M17 Gonarthrosis [arthrosis of knee]
M19 Other arthrosis
M20 Acquired deformities of fingers and toes
M23 Internal derangement of knee
M25 Other joint disorders, not elsewhere classified
M35 Other systemic involvement of connective tissue
M43 Other deforming dorsopathies
M47 Spondylosis
M48 Other spondylopathies
M51 Other intervertebral disk disorders
M54 Dorsalgia
M65 Synovitis and tenosynovitis
M67 Other disorders of synovium and tendon
M75 Shoulder lesions
M79 Other soft tissue disorders, not elsewhere classified
M81 Osteoporosis without pathological fracture
M85 Other disorders of bone density and structure
N13 Obstructive and reflux uropathy
N17 Acute renal failure
N18 Chronic renal failure
N20 Calculus of kidney and ureter
N28 Other disorders of kidney and ureter, not elsewhere classified
N30 Cystitis
N32 Other disorders of bladder
N39 Other disorders of urinary system
N73 Other female pelvic inflammatory diseases
N80 Endometriosis
N81 Female genital prolapse
N83 Noninflammatory disorders of ovary, Fallopian tube and broad ligament
N84 Polyp of female genital tract
N85 Other noninflammatory disorders of uterus, except cervix
N88 Other noninflammatory disorders of cervix uteri
N90 Other noninflammatory disorders of vulva and perineum
N92 Excessive, frequent and irregular menstruation
N93 Other abnormal uterine and vaginal bleeding
N94 Pain and other conditions associated with female genital organs and menstrual cycle
N95 Menopausal and other perimenopausal disorders
O68 Labour and delivery complicated by foetal stress [distress]
O70 Perineal laceration during delivery
<1 year
[1,5) years
>= 5 years

b

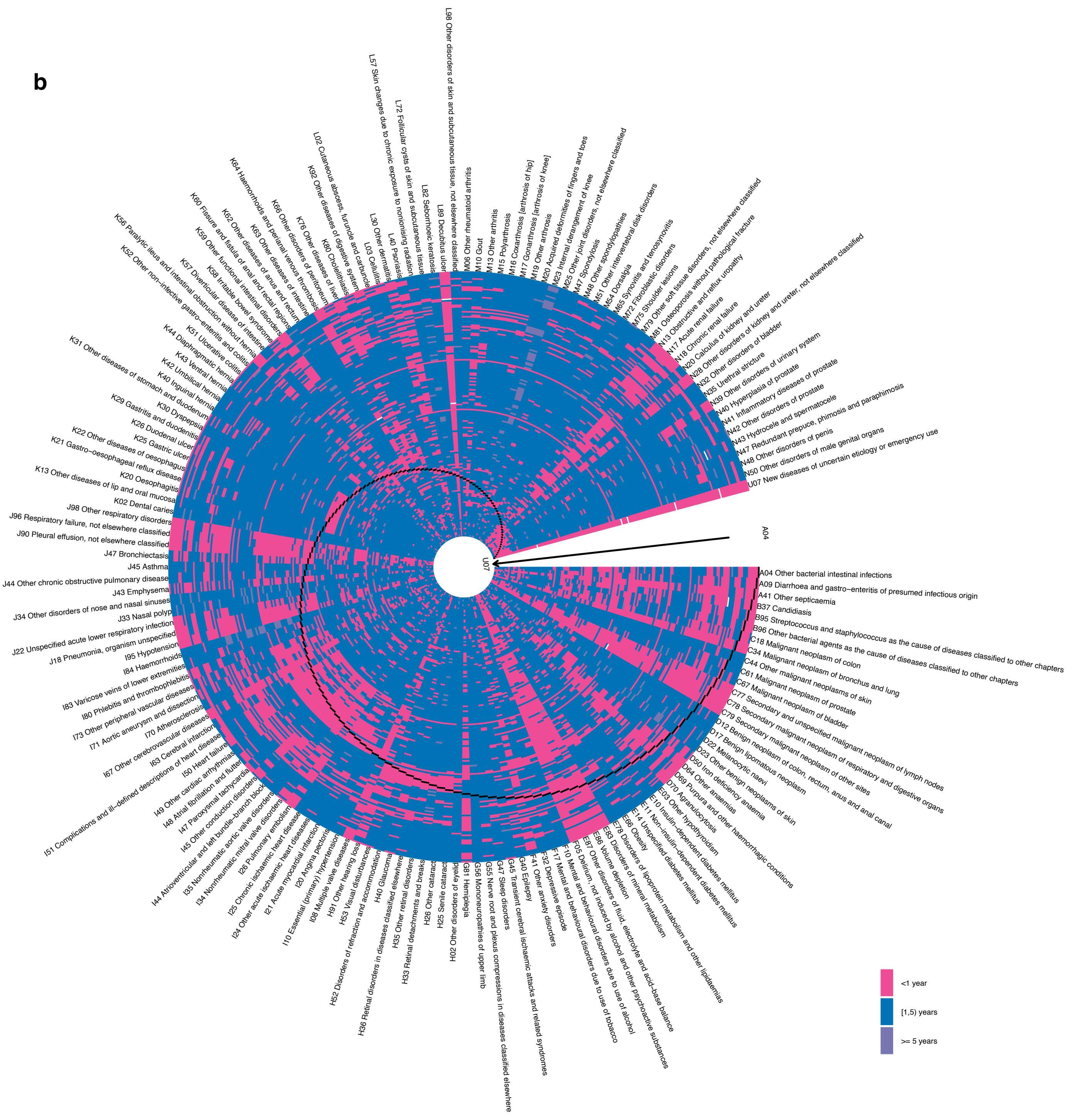

Supplementary Figure 6: **Median number of years between the onset of diseases studied**. **a**, females, median years of 23,562 (154 x 153) directional pairs. **b**, males, median years of 25,440 (160 x 159) directional pairs. Each cell plotted the median years for a directional pair, indicating the median of time interval from a disease on the outer circle to a disease on the radius among those who developed the disease on the outer circle and subsequently the disease on the radius during the study period. The majority of median years fall in the range of 1 to 5 years. Minimal and Maximal time intervals are shown in Supplementary Data 4 and 5. Cells in black represent diseases to themselves.

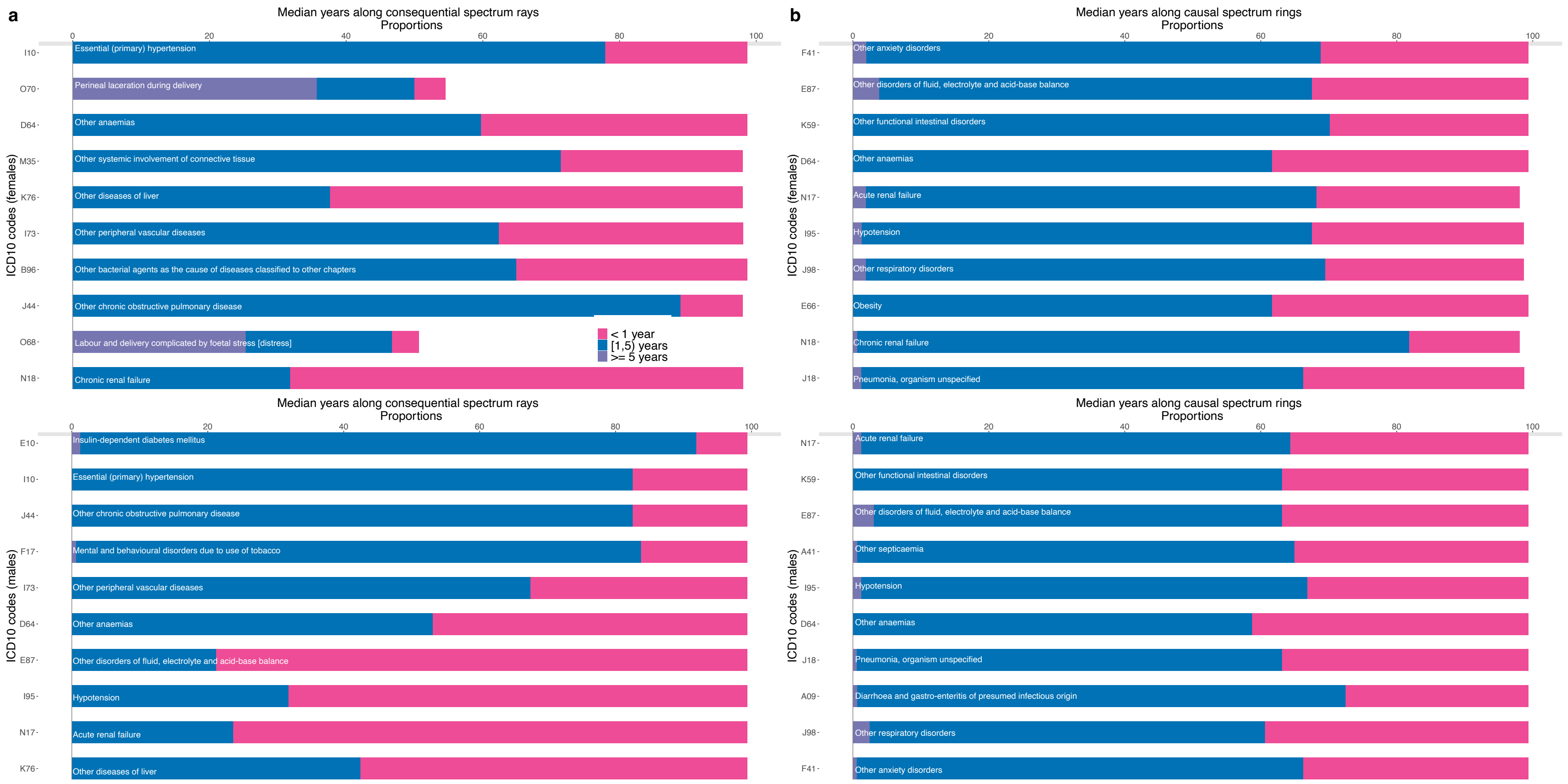

Supplementary Figure 7: **Distributions of the median number of years between the onset of top diseases and other diseases**. **a**, Between the top 10 influential diseases and their subsequent diseases. **b**, Between the top 10 influenced diseases and their previous diseases. Proportions may not total up to 100% as lack of corresponding samples during the study period.

a

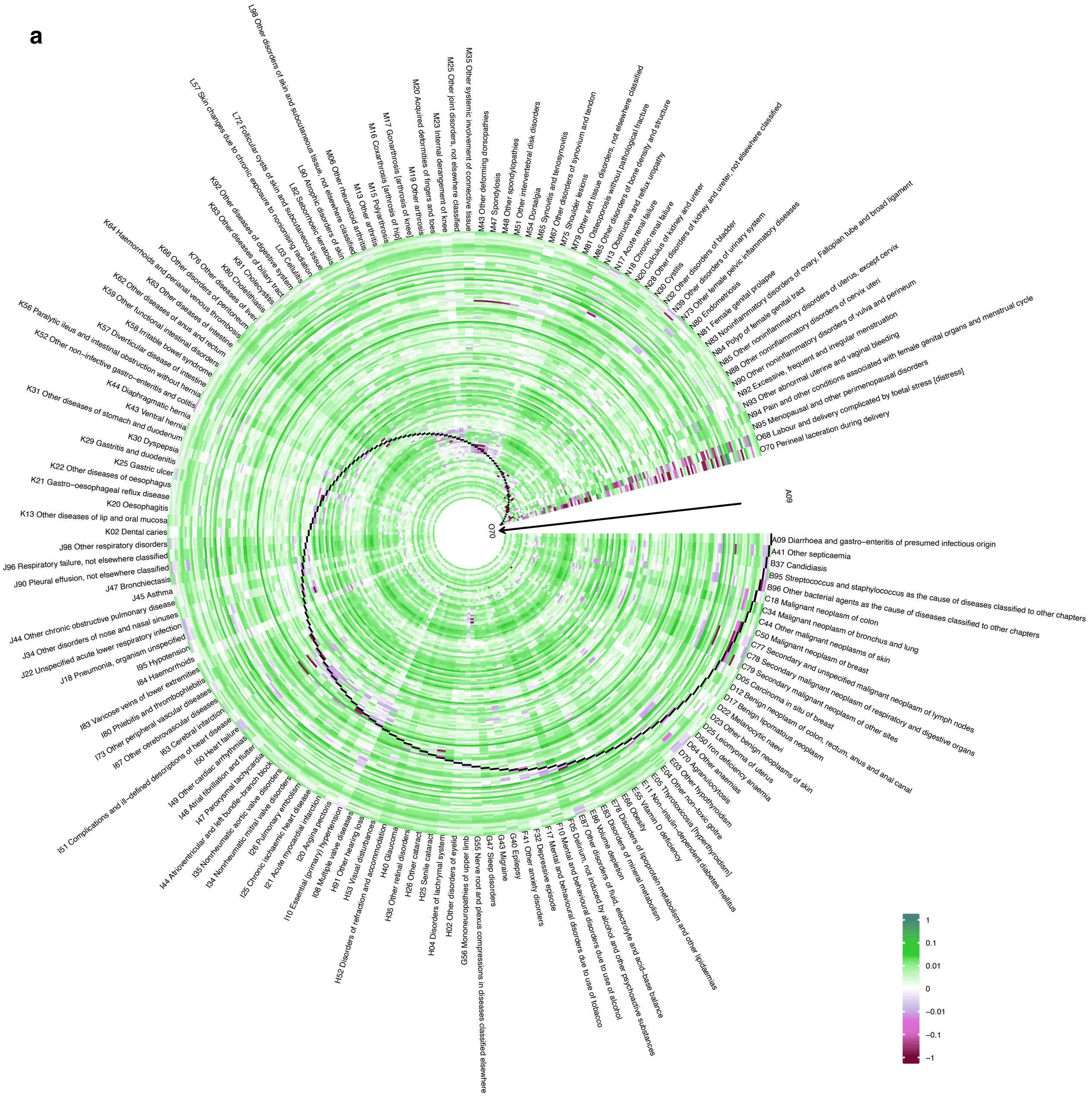
A09
O70
A09 Diarrhoea and gastro−enteritis of presumed infectious origin
A41 Other septicaemia
B37 Candidiasis
B95 Streptococcus and staphylococcus as the cause of diseases classified to other chapters
B96 Other bacterial agents as the cause of diseases classified to other chapters
C18 Malignant neoplasm of colon
C34 Malignant neoplasm of bronchus and lung
C44 Other malignant neoplasms of skin
C50 Malignant neoplasm of breast
C77 Secondary and unspecified malignant neoplasm of lymph nodes
C78 Secondary malignant neoplasm of respiratory and digestive organs
C79 Secondary malignant neoplasm of other sites
D05 Carcinoma in situ of breast
D12 Benign neoplasm of colon, rectum, anus and anal canal
D17 Benign lipomatous neoplasm
D22 Melanocytic naevi
D23 Other benign neoplasms of skin
D25 Leiomyoma of uterus
D50 Iron deficiency anaemia
D64 Other anaemias
D70 Agranulocytosis
E03 Other hypothyroidism
E04 Other non−toxic goitre
E05 Thyrotoxicosis [hyperthyroidism]
E11 Non−insulin−dependent diabetes mellitus
E55 Vitamin D deficiency
E66 Obesity
E78 Disorders of lipoprotein metabolism and other lipidaemias
E83 Disorders of mineral metabolism
E86 Volume depletion
E87 Other disorders of fluid, electrolyte and acid−base balance
F05 Delirium, not induced by alcohol and other psychoactive substances
F10 Mental and behavioural disorders due to use of alcohol
F17 Mental and behavioural disorders due to use of tobacco
F32 Depressive episode
F41 Other anxiety disorders
G40 Epilepsy
G43 Migraine
G47 Sleep disorders
G55 Nerve root and plexus compressions in diseases classified elsewhere
G56 Mononeuropathies of upper limb
H02 Other disorders of eyelid
H04 Disorders of lachrymal system
H25 Senile cataract
H26 Other cataract
H35 Other retinal disorders
H40 Glaucoma
H52 Disorders of refraction and accommodation
H53 Visual disturbances
H91 Other hearing loss
I08 Multiple valve diseases
I10 Essential (primary) hypertension
I20 Angina pectoris
I21 Acute myocardial infarction
I25 Chronic ischaemic heart disease
I26 Pulmonary embolism
I34 Nonrheumatic mitral valve disorders
I35 Nonrheumatic aortic valve disorders
I44 Atrioventricular and left bundle−branch block
I47 Paroxysmal tachycardia
I48 Atrial fibrillation and flutter
I49 Other cardiac arrhythmias
I50 Heart failure
I51 Complications and ill−defined descriptions of heart disease
I63 Cerebral infarction
I67 Other cerebrovascular diseases
I73 Other peripheral vascular diseases
I80 Phlebitis and thrombophlebitis
I83 Varicose veins of lower extremities
I84 Haemorrhoids
I95 Hypotension
J18 Pneumonia, organism unspecified
J22 Unspecified acute lower respiratory infection
J34 Other disorders of nose and nasal sinuses
J44 Other chronic obstructive pulmonary disease
J45 Asthma
J47 Bronchiectasis
J90 Pleural effusion, not elsewhere classified
J96 Respiratory failure, not elsewhere classified
J98 Other respiratory disorders
K02 Dental caries
K13 Other diseases of lip and oral mucosa
K20 Oesophagitis
K21 Gastro−oesophageal reflux disease
K22 Other diseases of oesophagus
K25 Gastric ulcer
K29 Gastritis and duodenitis
K30 Dyspepsia
K31 Other diseases of stomach and duodenum
K43 Ventral hernia
K44 Diaphragmatic hernia
K52 Other non−infective gastro−enteritis and colitis
K56 Paralytic ileus and intestinal obstruction without hernia
K57 Diverticular disease of intestine
K58 Irritable bowel syndrome
K59 Other functional intestinal disorders
K62 Other diseases of anus and rectum
K63 Other diseases of intestine
K64 Haemorrhoids and perianal venous thrombosis
K66 Other disorders of peritoneum
K76 Other diseases of liver
K80 Cholelithiasis
K81 Cholecystitis
K83 Other diseases of biliary tract
K92 Other diseases of digestive system
L03 Cellulitis
L57 Skin changes due to chronic exposure to nonionising radiation
L72 Follicular cysts of skin and subcutaneous tissue
L82 Seborrhoeic keratosis
L90 Atrophic disorders of skin
L98 Other disorders of skin and subcutaneous tissue, not elsewhere classified
M06 Other rheumatoid arthritis
M13 Other arthritis
M15 Polyarthrosis
M16 Coxarthrosis [arthrosis of hip]
M17 Gonarthrosis [arthrosis of knee]
M19 Other arthrosis
M20 Acquired deformities of fingers and toes
M23 Internal derangement of knee
M25 Other joint disorders, not elsewhere classified
M35 Other systemic involvement of connective tissue
M43 Other deforming dorsopathies
M47 Spondylosis
M48 Other spondylopathies
M51 Other intervertebral disc disorders
M54 Dorsalgia
M65 Synovitis and tenosynovitis
M67 Other disorders of synovium and tendon
M75 Shoulder lesions
M79 Other soft tissue disorders, not elsewhere classified
M81 Osteoporosis without pathological fracture
M85 Other disorders of bone density and structure
N13 Obstructive and reflux uropathy
N17 Acute renal failure
N18 Chronic renal failure
N20 Calculus of kidney and ureter
N28 Other disorders of kidney and ureter, not elsewhere classified
N30 Cystitis
N32 Other disorders of bladder
N39 Other disorders of urinary system
N73 Other female pelvic inflammatory diseases
N80 Endometriosis
N81 Female genital prolapse
N83 Noninflammatory disorders of ovary, Fallopian tube and broad ligament
N84 Polyp of female genital tract
N85 Other noninflammatory disorders of uterus, except cervix
N88 Other noninflammatory disorders of cervix uteri
N90 Other noninflammatory disorders of vulva and perineum
N92 Excessive, frequent and irregular menstruation
N93 Other abnormal uterine and vaginal bleeding
N94 Pain and other conditions associated with female genital organs and menstrual cycle
N95 Menopausal and other perimenopausal disorders
O68 Labour and delivery complicated by foetal stress [distress]
O70 Perineal laceration during delivery
1
0.1
0.01
0
−0.01
−0.1
−1

b

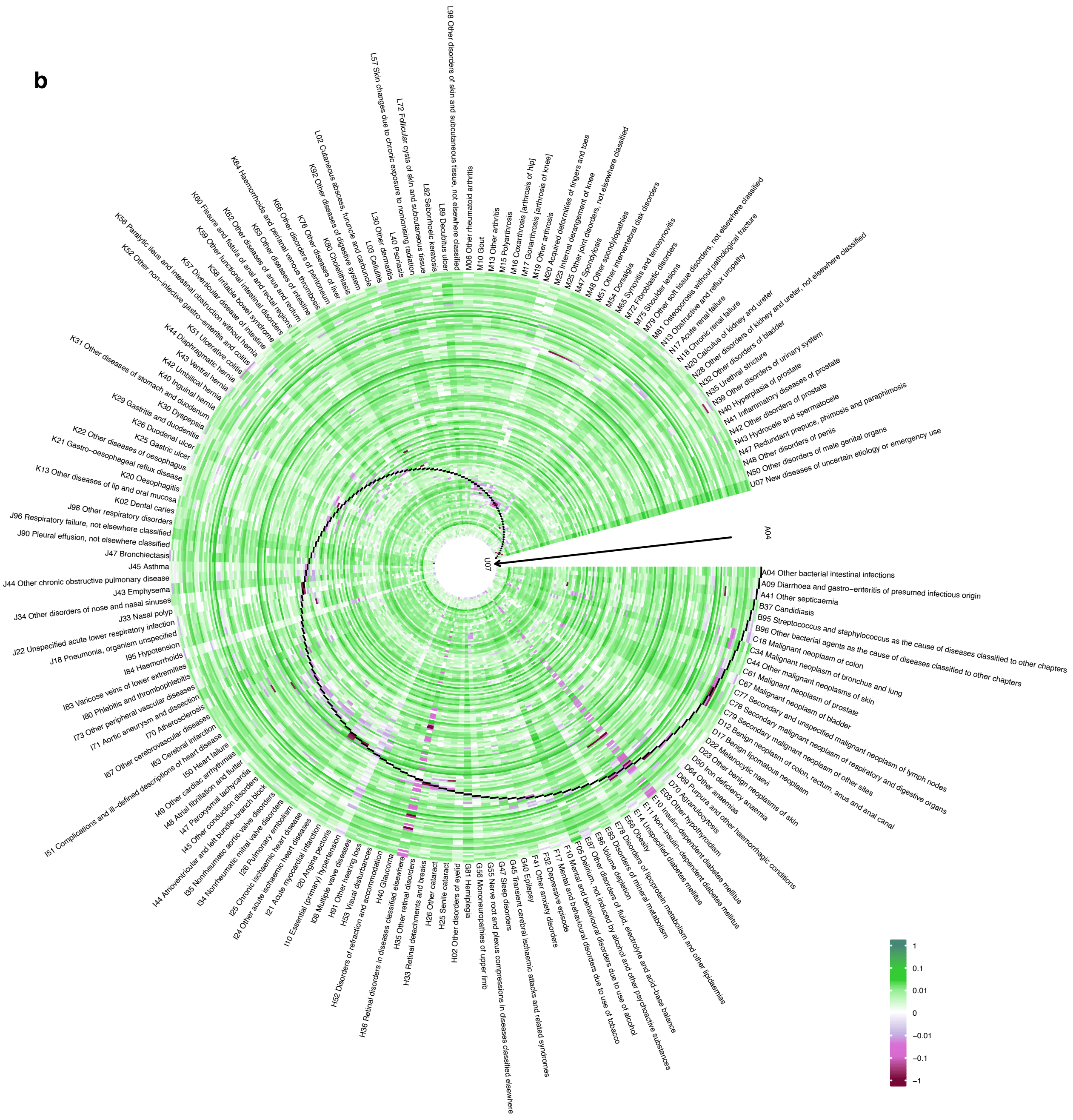

Supplementary Figure 8: Changes in pairwise causal effects between the main analysis and the secondary analysis with one year of follow-up. **a**, females, changes in estimates of 23,562 (154 x 153) directional pairwise causal effects of diseases. **b**, males, changes in estimates of 25,440 (160 x 159) directional pairwise causal effects of diseases. Each cell plotted the differences in the estimated causal effects of a directional pair. Cells in black represent diseases to themselves.

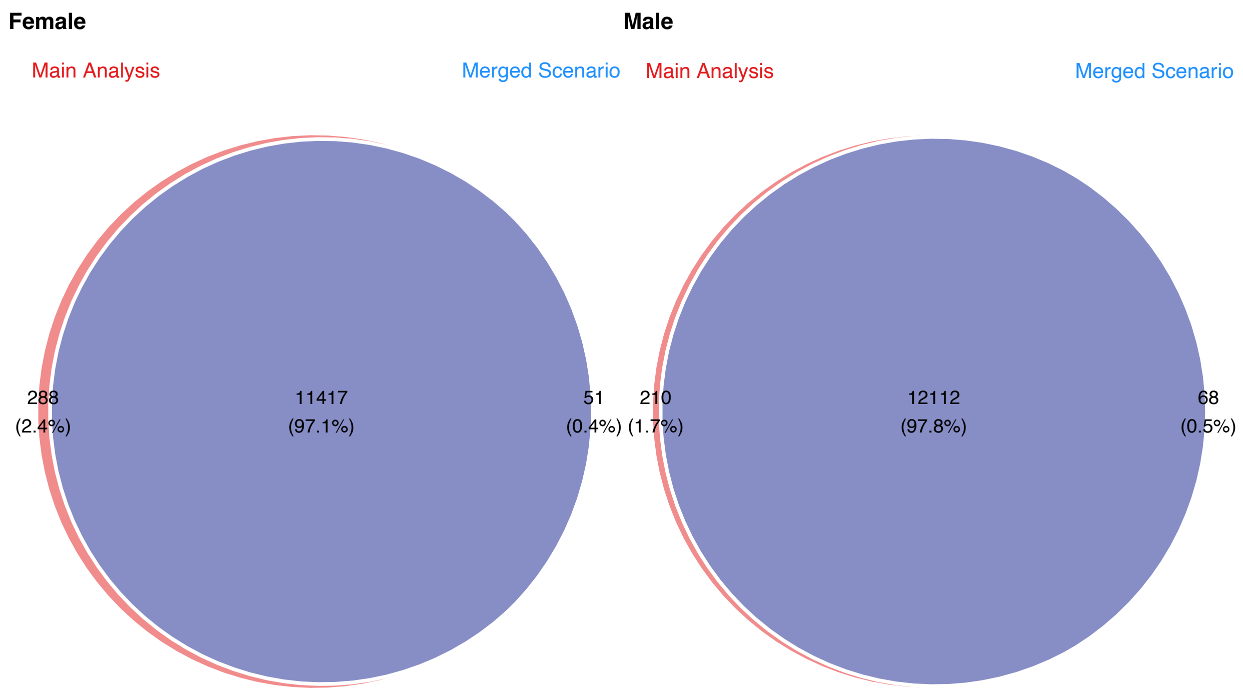

Supplementary Figure 9: Venn diagrams show the overlap of identified pairs with significant causal effects between the main analysis and merged scenario.

Supplementary Tables

Supplementary Table 1. Detailed baseline variables in UKBB

Num	Category	Field ID	Field Name
1	Alcohol	1558	Alcohol intake frequency
2	Baseline characteristics	31	Sex
		189	Townsend deprivation index at recruitment
		21022	Age at recruitment
3	Blood biochemistry	30680	Calcium
		30710	C-reactive protein
		30750	Glycated haemoglobin (HbA1c)
		30760	High Density Lipoprotein (HDL) cholesterol
		30780	Low Density Lipoprotein (LDL) direct
		30870	Triglycerides
4	Blood pressure	4079	Diastolic blood pressure, automated reading
		4080	Systolic blood pressure, automated reading
5	Body size measures	21001	Body mass index (BMI)
6	Education	6138	Qualifications
7	Electronic device use	1120	Weekly usage of mobile phone in last 3 months
8	Ethnicity	21000	Ethnic background
9	Family history	1807	Father's age at death
		3526	Mother's age at death
		20107	Illnesses of father
		20110	Illnesses of mother
10	General health	2188	Long-standing illness, disability or infirmity
11	Household	6139	Gas or solid-fuel cooking/heating
12	Metabolic Equivalent Task (MET) Scores	22032	International Physical Activity Questionnaire (IPAQ) activity group
13	Sleep	1160	Sleep duration
14	Smoking	1239	Current tobacco smoking
15	Social support	6160	Leisure/social activities
16	Summary Diagnosis	41270	Diagnoses - ICD10

References

1. Schuler MS, Rose S. Targeted Maximum Likelihood Estimation for Causal Inference in Observational Studies. *American Journal of Epidemiology*. 2017;185(1):65-73.
2. Azur MJ, Stuart EA, Frangakis C, Leaf PJ. Multiple imputation by chained equations: what is it and how does it work? *International Journal of Methods in Psychiatric Research*. 2011;20(1):40.
3. Rubin DB. *Multiple Imputation for Nonresponse in Surveys*. John Wiley & Sons, Ltd; 2004.
4. Stürmer T, Webster-Clark M, Lund JL, et al. Propensity Score Weighting and Trimming Strategies for Reducing Variance and Bias of Treatment Effect Estimates: A Simulation Study. *American Journal of Epidemiology*. 2021;190(8):1659-1670.
5. Defays D. An efficient algorithm for a complete link method. *The Computer Journal*. 1977;20(4):364-366.
6. Ward JH. Hierarchical Grouping to Optimize an Objective Function. *Journal of the American Statistical Association*. 1963;58(301):236-244.
7. Shimodaira H. An Approximately Unbiased Test of Phylogenetic Tree Selection. *Systematic Biology*. 2002;51(3):492-508.
8. Hennig C, Liao TF. How to find an appropriate clustering for mixed-type variables with application to socio-economic stratification. *Journal of the Royal Statistical Society: Series C (Applied Statistics)*. 2013;62(3):309-369.
9. Hubert L, Arabie P. Comparing partitions. *Journal of Classification*. 1985;2(1):193-218.
10. Ng A, Jordan M, Weiss Y. On Spectral Clustering: Analysis and an algorithm. *Advances in Neural Information Processing Systems*. 2001;14.
11. Von Luxburg U. A tutorial on spectral clustering. *Statistics and Computing*. 2007;17(4):395-416.
12. Kamada T, Kawai S. An algorithm for drawing general undirected graphs. *Information Processing Letters*. 1989;31(1):7-15.
13. Brin S, Page L. The anatomy of a large-scale hypertextual Web search engine. *Computer Networks and ISDN Systems*. 1998;30(1-7):107-117.
