# Supplementary material for: Mapping multimorbidity progression among 190 diseases
Source: Commun Med (Lond). 2024 Jul 11;4:139. doi: 10.1038/s43856-024-00563-2 (PMC11239867; doi:10.1038/s43856-024-00563-2)
Supplement: Supplementary file 3 — Description of Additional Supplementary Files [file 43856_2024_563_MOESM3_ESM.pdf]

Description of Additional Supplementary Files

File name: Supplementary Data 1.

File Description: Pairwise causal effects of 154 diseases for females.

File name: Supplementary Data 2.

File Description: Pairwise causal effects of 160 diseases for males.

File name: Supplementary Data 3.

File Description: Significance of patterns of one-step progress among ICD-10 chapters by sex and progress direction.

File name: Supplementary Data 4.

File Description: Years of follow-up for female samples by pairs of diseases.

File name: Supplementary Data 5.

File Description: Years of follow-up for male samples by pairs of diseases.
