# Supplementary material for: Mapping multimorbidity progression among 190 diseases
Source: Commun Med (Lond). 2024 Jul 11;4:139. doi: 10.1038/s43856-024-00563-2 (PMC11239867; doi:10.1038/s43856-024-00563-2)
Supplement: Supplementary file 9 — Reporting summary [file 43856_2024_563_MOESM9_ESM.pdf]

nature portfolio

Corresponding author(s): Shasha Han, Chen Wang

Last updated by author(s): Jun 5, 2024

Reporting Summary

Nature Portfolio wishes to improve the reproducibility of the work that we publish. This form provides structure for consistency and transparency in reporting. For further information on Nature Portfolio policies, see our Editorial Policies and the Editorial Policy Checklist.

Statistics

For all statistical analyses, confirm that the following items are present in the figure legend, table legend, main text, or Methods section.

n/a	Confirmed	
☐	☒	The exact sample size (*n*) for each experimental group/condition, given as a discrete number and unit of measurement
☐	☒	A statement on whether measurements were taken from distinct samples or whether the same sample was measured repeatedly
☐	☒	The statistical test(s) used AND whether they are one- or two-sided<br>*Only common tests should be described solely by name; describe more complex techniques in the Methods section.*
☐	☒	A description of all covariates tested
☐	☒	A description of any assumptions or corrections, such as tests of normality and adjustment for multiple comparisons
☐	☒	A full description of the statistical parameters including central tendency (e.g. means) or other basic estimates (e.g. regression coefficient) AND variation (e.g. standard deviation) or associated estimates of uncertainty (e.g. confidence intervals)
☐	☒	For null hypothesis testing, the test statistic (e.g. *F*, *t*, *r*) with confidence intervals, effect sizes, degrees of freedom and *P* value noted<br>*Give P values as exact values whenever suitable.*
☒	☐	For Bayesian analysis, information on the choice of priors and Markov chain Monte Carlo settings
☒	☐	For hierarchical and complex designs, identification of the appropriate level for tests and full reporting of outcomes
☒	☐	Estimates of effect sizes (e.g. Cohen's *d*, Pearson's *r*), indicating how they were calculated

Our web collection on statistics for biologists contains articles on many of the points above.

Software and code

Policy information about availability of computer code

Data collection	R 4.3.0 was used to perform collation and analyses.
Data analysis	R 4.3.0 was used to perform the statistical analyses and present the results. The code used to analyze the data is available at https://github.com/ShashaHan-collab/MapMultimorbidityCluster

For manuscripts utilizing custom algorithms or software that are central to the research but not yet described in published literature, software must be made available to editors and reviewers. We strongly encourage code deposition in a community repository (e.g. GitHub). See the Nature Portfolio guidelines for submitting code & software for further information.

Data

Policy information about availability of data

All manuscripts must include a data availability statement. This statement should provide the following information, where applicable:

- Accession codes, unique identifiers, or web links for publicly available datasets
- A description of any restrictions on data availability
- For clinical datasets or third party data, please ensure that the statement adheres to our policy

Data was available from the can be requested directly from the UK Biobank project site, after successful completion of registration and the application process. Full details can be found https://www.ukbiobank.ac.uk/. Source data are provided at https://github.com/ShashaHan-collab/MapMultimorbidityCluster

Human research participants

Policy information about studies involving human research participants and Sex and Gender in Research.

Reporting on sex and gender	The findings apply to both females and males. Sex was considered in the study design and was determined based on self-report.<br>Disaggregated data on sex were collected, and consent was obtained for reporting and sharing individual-level data.<br>Disaggregated data on sex was provided in the source data. There were 502,413 participants at baseline, of whom 54.4% were women.<br>Sex-based analyses were performed and reported in the manuscript as follows:<br>"All the analyses were conducted separately for females and males to compare multimorbidity progress between sexes."
Population characteristics	See above.
Recruitment	The UKB is a multicenter, community-based cohort study conducted in the United Kingdom from March 2006 to December 2010 for 502,413 participants at baseline, of whom 54.4% were women (aged 37 to 73 years, mean[SD] age 57.1[8.1] years). All participants were registered with the UK National Health Service and attended an initial examination, and were followed up longitudinally through linkage to electronic health-related records. Ongoing inpatient hospital records beginning in 1995 until September 2021 were used to identify diagnoses according to the International Statistical Classification of Diseases and Related Health Problems, Tenth Revision (ICD-10) codes. End of follow-up was defined as end of hospital inpatient data collection in September 2021; mean longitudinal follow-up was 12.7 [0.9] years.<br><br>The study is an observational study, which can be subject to potential confounding bias. While we have made every effort to control for measured confounders and found significant effects between many pairs of diseases, alternative explanations are possible. Additionally, there could be unmeasured shared etiology that may also be responsible for the observed causal links that are not accounted for in our data, such as diseases that occurred in early childhood, unmeasured environmental exposures, and common genetically determined mechanisms.
Ethics oversight	This study was covered by the ethical approval from the UKB granted by the National Information Governance Board for Health and Social Care and the NHS North West Multicenter Research Ethics Committee. All participants provided informed consent through electronic signature at baseline assessment. Ethical approval of the study was obtained from the Chinese Academy of Medical Sciences & Peking Union Medical College. All data extracted were deidentified for analysis.

Note that full information on the approval of the study protocol must also be provided in the manuscript.

Field-specific reporting

Please select the one below that is the best fit for your research. If you are not sure, read the appropriate sections before making your selection.

☒ Life sciences ☐ Behavioural & social sciences ☐ Ecological, evolutionary & environmental sciences

For a reference copy of the document with all sections, see nature.com/documents/nr-reporting-summary-flat.pdf

Life sciences study design

All studies must disclose on these points even when the disclosure is negative.

Sample size	This cohort study was carried out on 502,413 adult participants from UK Biobank.
Data exclusions	No data were excluded from analyses.
Replication	(1) A sensitivity analysis was conducted to investigate whether the main conclusions remained after accounting for variations across different imputations. P values were subsequently adjusted for multiple testing based on false discovery rate using Benjamini and Hochberg method. We compared the number of significant causal effects (adjusted P values < 0.01) for the main analysis and the merged scenarios. The results showed that over 97% of the causal pairs were identified in both methods, for both females and males (Supplementary Fig. 1). (2) As an alternative analysis, we tested the stability by comparing results across distinct clustering methods. We found that the adjusted Rand Indexes were still sufficiently high, 0.643 and 0.679 for clusterings in females and males respectively.
Randomization	This is an observational study, and allocation was not random. We used causal inference method to balance covariates between groups.
Blinding	NA. This is an observational study.

Reporting for specific materials, systems and methods

We require information from authors about some types of materials, experimental systems and methods used in many studies. Here, indicate whether each material, system or method listed is relevant to your study. If you are not sure if a list item applies to your research, read the appropriate section before selecting a response.

Materials & experimental systems

n/a	Involved in the study
☒	☐ Antibodies
☒	☐ Eukaryotic cell lines
☒	☐ Palaeontology and archaeology
☒	☐ Animals and other organisms
☒	☐ Clinical data
☒	☐ Dual use research of concern

Methods

n/a	Involved in the study
☒	☐ ChIP-seq
☒	☐ Flow cytometry
☒	☐ MRI-based neuroimaging
